# Isolation of a member of the candidate phylum ‘Atribacteria’ reveals a unique cell membrane structure

**DOI:** 10.1038/s41467-020-20149-5

**Published:** 2020-12-14

**Authors:** Taiki Katayama, Masaru K. Nobu, Hiroyuki Kusada, Xian-Ying Meng, Naoki Hosogi, Katsuyuki Uematsu, Hideyoshi Yoshioka, Yoichi Kamagata, Hideyuki Tamaki

**Affiliations:** 1 Geomicrobiology Research Group, Research Institute for Geo-Resources and Environment, Geological Survey of Japan, National Institute of Advanced Industrial Science and Technology (AIST), Central 7, 1-1-1 Higashi, Tsukuba, Ibaraki 305-8567 Japan; 2 Bioproduction Research Institute, Central 6-10, 1-1-1 Higashi, AIST, Tsukuba, Ibaraki 305-8566 Japan; 3 EM Application Department, EM Business Unit, JEOL, Ltd., 3-1-2 Musashino, Akishima, Tokyo 196-8558 Japan; 4 Department of Marine and Earth Sciences, Marine Work Japan Ltd., 3-54-1 Oppamahigashi, Yokosuka, Kanagawa 237-0063 Japan

**Keywords:** Cell biology, Bacterial physiology, Cellular microbiology

## Abstract

A key feature that differentiates prokaryotic cells from eukaryotes is the absence of an intracellular membrane surrounding the chromosomal DNA. Here, we isolate a member of the ubiquitous, yet-to-be-cultivated phylum ‘*Candidatus* Atribacteria’ (also known as OP9) that has an intracytoplasmic membrane apparently surrounding the nucleoid. The isolate, RT761, is a subsurface-derived anaerobic bacterium that appears to have three lipid membrane-like layers, as shown by cryo-electron tomography. Our observations are consistent with a classical gram-negative structure with an additional intracytoplasmic membrane. However, further studies are needed to provide conclusive evidence for this unique intracellular structure. The RT761 genome encodes proteins with features that might be related to the complex cellular structure, including: N-terminal extensions in proteins involved in important processes (such as cell-division protein FtsZ); one of the highest percentages of transmembrane proteins among gram-negative bacteria; and predicted Sec-secreted proteins with unique signal peptides. Physiologically, RT761 primarily produces hydrogen for electron disposal during sugar degradation, and co-cultivation with a hydrogen-scavenging methanogen improves growth. We propose RT761 as a new species, *Atribacter laminatus* gen. nov. sp. nov. and a new phylum, *Atribacterota* phy. nov.

## Abstract (web summary)

A key feature that differentiates prokaryotic cells from eukaryotes is the absence of an intracellular membrane surrounding the chromosomal DNA. Here, the authors isolate a member of the ubiquitous, yet-to-be-cultivated bacterial phylum ‘*Candidatus* Atribacteria’ that has an intracytoplasmic membrane apparently surrounding the nucleoid.

## Introduction

Cultivation of uncultured microorganisms is a critical step in uncovering their phenotypic features, such as cell structure and metabolic function. However, most lineages of the domains *Bacteria* and *Archaea* remain uncharacterized^1^ due to difficulties in cultivation^2,3^. While omics-based cultivation-independent characterization can circumvent cultivation and provide insight into metabolism and ecology^4,5^, metabolic reconstruction is generally based on genes characterized in cultured organisms and, thus, prediction of novel phenotypic features of uncultured microorganisms remains challenging^6^.

In this study, we have isolated an anaerobic bacterium that belongs to the bacterial candidate phylum “*Ca*. Atribacteria”^4^ (“*Ca*. Caldatribacteriota” in Genome Taxonomy Database^7^). “*Ca*. Atribacteria” was originally discovered through 16S rRNA gene clones in sediments from the hot spring in Yellowstone National Park and designated as OP9 in 1998^8^, and recently proposed to include the JS1 lineage^5^. The members of this phylum are globally distributed and, in some cases abundant, in anaerobic environments^5^. Here, we report structural, genomic and physiological characterization of the cultured representative of “*Ca*. Atribacteria”.

## Results and discussion

### Multilayered cell structure

**Fig. 1:**
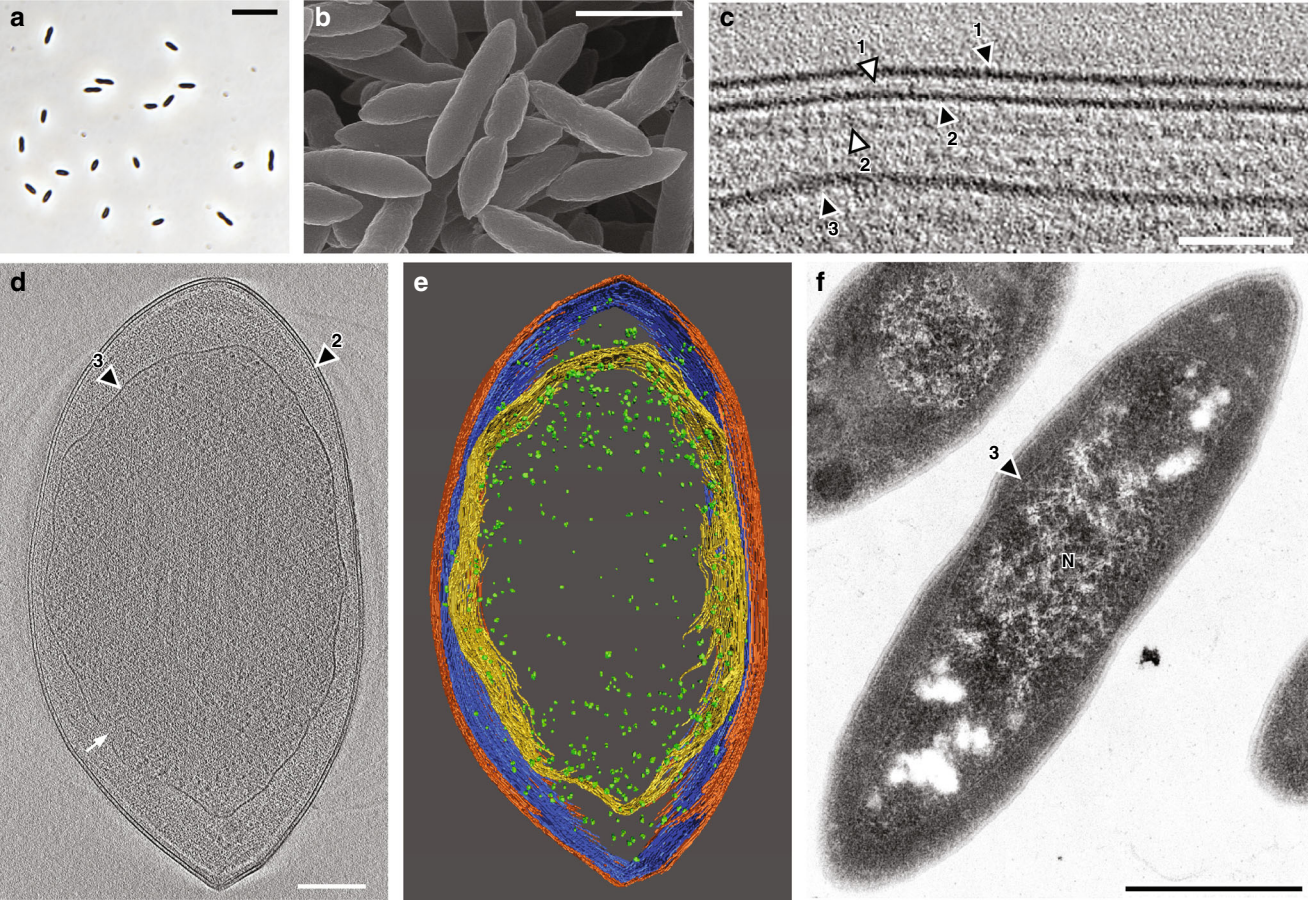
Morphology and membrane structure in RT761 cells showing the presence of three lipid membrane-like layers (LMLs) with the innermost layer surrounding the nucleoid. **a** Phase-contrast microscopy. **b** Scanning electron microscopy. **c**–**e** Cryo-electron tomography (also see Supplementary Movies 1 and 2). **c** The original slice picture is shown in Supplementary Fig. 4. Black arrowheads indicate the outer (1), middle (2), and inner (3) LMLs. White arrowheads indicate the 2.2 nm thick layer (1) and faint layers (2). **d** White arrow indicates inner LML invagination. **e** 3D-rendered reconstruction of the cell in **d**. (also see Supplementary Movie 4). Color code: outer LML, orange; middle LML, blue; inner LML, yellow; ribosome, green. **f** Transmission electron micrograph of a thin section of RT761 cells. N nucleoid. Scale bars, 5.0 μm (**a**), 1.5 μm (**b**), 0.2 μm (**c**), 0.1 μm (**d**, **e**), and 0.5 μm (**f**).

**Fig. 2:**
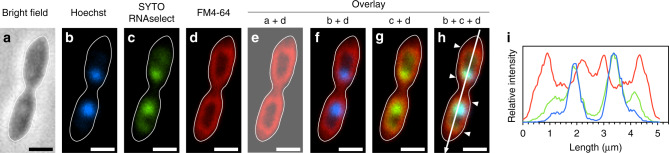
Confocal-laser microscopy showing the localization of DNA and RNA within the intracytoplasmic membrane structure. DNA, RNA, and membrane lipids were stained by Hoechst (blue), SYTO RNAselect (green) and FM4-64 (red), respectively. Outlines of the cell from **a** are included in all panels. **a** Phase contrast image. **b**–**d** Confocal-laser images. **e**–**h** Image overlays. **i** Line profiles of fluorescence intensity plotted longitudinally along white arrow in **h**. Membrane staining maxima are indicated by arrowheads in **h**. Source data are provided as a Source Data file. Scale bars, 1 μm.

A bacterium, designated RT761, was isolated after 3 years of enrichment from saline formation water and sediments derived from deep aquifers in natural-gas deposits in Japan. Phylogenetic analysis based on 16S rRNA gene and conserved protein-coding markers revealed that strain RT761 was assigned to the clade OP9 of “*Ca*. Atribacteria” (Supplementary Figs. 1 and 2). RT761 cells are Gram-stained negative, nonspore forming, and tapered rod or ovoid-shaped with pointed ends (Fig. 1a, b). Although typical gram-negative cells only have two lipid bilayers (cytoplasmic membrane [CM] and outer membrane [OM]), cryo-electron tomography (CET) revealed that RT761 possesses three layers with appearances characteristic of lipid bilayers: a 4 nm thickness and two leaflets (Fig. 1c, d and Supplementary Figs. 3, 4 and Supplementary Movies 1 and 2). Between the outer lipid membrane-like layer (LML) and middle LML, we observe a narrow space (7.0 nm [SD 0.3]) with a thin layer of 2.2 nm width (SD 0.1) (Fig. 1c and Supplementary Fig. 4). The outer LML was occasionally observed (15 out of 31 cells) to have small embedded jagged structures (periodicity of ca. 4 nm) comprising between 1.9 and 13.7% of cell outline in two-dimensional images (Supplementary Figs. 3, 4 and Supplementary Movie 3). The inner LML has an amorphous structure (including occasional small invaginations) that does not follow the inside of the other LML, and is likely separated from the other LMLs (Fig. 1d, e and Supplementary Movies 1, 2, 4). We observe ribosomes in both the inner LML-bounded space [19.8 nm diameter (SD 0.7)] and middle LML-bounded space [20.1 nm (SD 0.8)] (Supplementary Fig. 5). More are observed to be present in the former based on CET and fluorescence microscopy (Supplementary Figs. 5 and 6). The inner LML is most distant from the other membranes at the cell poles, creating large polar spaces (Fig. 1d, e). Additional observation through transmission electron microscopy shows that the inner LML appears to envelopes the RT761 nucleoid (Fig. 1f). This was further supported by the fluorescence microscopy-based observation of bulk DNA/RNA in the center of the cell bounded by a lipid membrane structure (stained with FM4-64), leaving open polar spaces (Fig. 2). (In some photomicrographs, the RT761 outer two LML may not be stained well by FM4-64 as, in the presence of multiple membrane layers, the dye can tend to stain outermost layers less^9–11^). During cell division, the three LML are observed to be clearly maintained at the site of binary fission, and the inner LML apparently continues to surround the nucleoid, invaginates following the two other LML (but does not directly attach), and splits into the daughter cells as division completes (Supplementary Figs. 7 and 8). At the site of division, we observe three distinct sets of layers, unlike typical gram-negative bacteria (Supplementary Fig. 7). These observations suggest that the three LMLs are not connected to each other. While the exact identity/composition of the three LMLs remain unclear, the results point to two possibilities. The jagged structures in the outer LML suggest that the outer layer may be proteinaceous, in which case, the layers would represent a surface protein layer (outer LML), OM (middle LML), and CM (inner LML). On the other hand, the lipid bilayer-like characteristics of the observed layers suggest RT761 possesses three lipid membranes, in which case, the observed layers would represent the OM (outer LML with high protein density like *Thermotoga*^12^), peptidoglycan layer (2.2 nm thick layer), CM (middle LML), and a intracytoplasmic membrane (inner LML) surrounding the cell’s nucleoid.

### Unique genomic features

The potential importance of localization and membranes in RT761 was supported by genomic and transcriptomic analyses. Alignment of all RT761 protein-coding genes with a reference sequence database revealed that 34 genes in the RT761 genome contained unique N-terminal extensions (NTE; 10–73 amino acids in length) compared to the top 250 hits in the NCBI RefSeq database and some of them were conserved among “*Ca*. Atribacteria” OP9 genomes (Supplementary Datas 1 and 2). The genes with NTE included those involved in critical cellular processes: cell division (FtsZ), Lipid A biosynthesis (UDP-3-O-acyl-*N*-acetyglucosamine deacetylase—LpxC), DNA replication, DNA repair, transcriptional regulation, tRNA processing, transmembrane signaling, and H_2_ generation (FeFe hydrogenase subunit alpha—HydA). Notably, several facilitate central functions in their respective processes: FtsZ recruits other cell division proteins to the fission site^13^, LpxC performs the committing step in Lipid A biosynthesis^14^, and HydA catalyzes the reduction of protons to H_2_ in the hydrogenase complex^15^. NTEs in prokaryotes have so far been only found in enzymes that localize to the lumen of subcellular compartments called bacterial microcompartment (BMC), and are necessary for the encapsulation of enzymes with NTE into BMC shells^16,17^. Among 34 genes with NTE in RT761, only one enzyme (deoxyribose-phosphate aldolase) may be related to BMC because of its occurrence in the gene cluster encoding homologs of BMC (although BMC-like compounds were not visible in electron microscopy). Interestingly, RT761 and other “*Ca*. Atribacteria” possessed two FtsZ genes—one conventional FtsZ and another with an NTE that is predicted to form an amphipathic helix (Supplementary Fig. 9). RT761 expressed both FtsZ genes (RT761_00112 and RT761_02154) during exponential growth (Supplementary Data 3). While the NTE-lacking FtsZ gene is adjacent to FtsA, the NTE-possessing FtsZ lacks a corresponding FtsA (i.e., only one copy of *ftsA* in the genome). FtsA facilitates association between FtsZ and the CM through a C-terminal amphipathic helix^18^, suggesting that the NTE-possessing FtsZ may be capable of binding to the membrane. Although 9 out of 6751 cultured bacterial type strains possess both a typical and NTE-possessing FtsZ, putative amphipathic helices were not found in any of these sequences. Such unique features may play key roles in binary fission through multi-layers in RT761 (Supplementary Figs. 7 and 8), but additional investigations are necessary for verification.

**Fig. 3:**
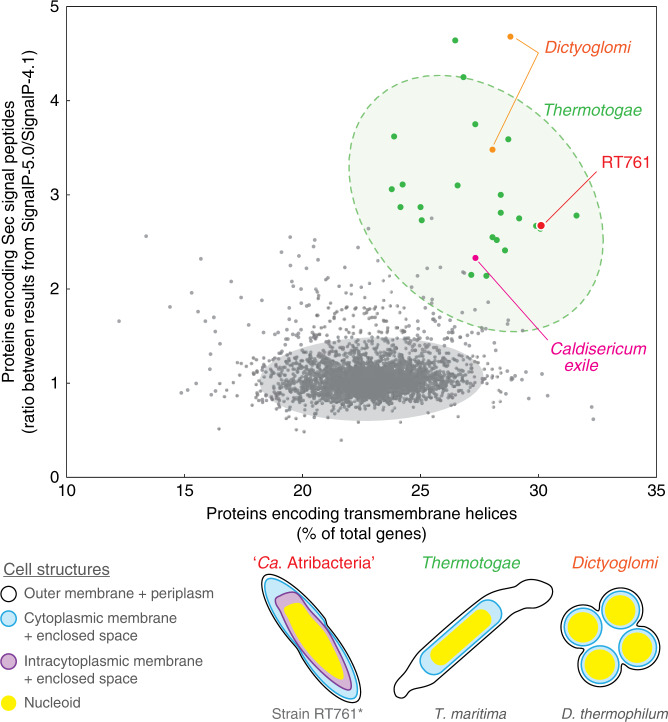
Unique genomic compositions of membrane-related features observed for phyla with unique cell structures. The horizontal axis shows the genomic proportion proteins encoding transmembrane helices. The vertical axis shows the ratio of proportions of proteins encoding Sec signal peptides estimated by SignalP-5.0 and SignalP-4.1. RT761 (red), type strains from *Thermotogae* (green), *Dictyoglomi* (orange), *Caldiserica* (pink), and other gram-negative type strains (gray) are plotted (3502 genomes downloadable from the Joint Genome Institute Integrated Microbial Genomes and Microbiomes database). For *Thermotogae* and other gram-negative type strains, 95% (green) and 99.9% (gray) confidence ellipses are shown respectively. Source data are provided as a Source Data file. (Bottom) Cell structures of select species are shown for “*Ca*. Atribacteria”, *Thermotogae*, and *Dictyoglomi*. The illustrations indicate the outer membrane (black), cytoplasmic membrane (blue), intracytoplasmic membrane (purple), and nucleoid (yellow). *For RT761, the shown schematic requires further investigation to conclude the identity/role of each layer.

Further analysis reveals unique genomic features of RT761 related to membrane-mediated physiology. Based on transcriptomic analysis of RT761 under exponential growth phase, membrane-associated proteins comprised 5 out of 10 of the mostly highly expressed genes (Supplementary Data 3). These include a putative transmembrane protein (RT761_00009), lipoprotein (RT761_00907), periplasmic substrate-binding protein (RT761_02219), and two fasciclin domain-containing transmembrane proteins (RT761_00748 and 01694), all of which have unknown functions. These findings point toward the importance of membrane-centric metabolism in RT761 physiology. The RT761 genome also has a high proportion of proteins with transmembrane helices (29.6% of all proteins) greater than 99.7% of all gram-negative type strains with sequenced genomes available (Fig. 3).

We also found that RT761 may have unique signal peptide sequences for Sec-secreted proteins. While SignalP-4.1^19^ estimated that RT761 has a low proportion of Sec-secreted proteins (3.4% of all proteins) less than 96.7% of all Gram-negative type strains, SignalP-5.0^20^ predicted 2.67 times more (9.0% of all proteins) (Fig. 3). Evaluation of all gram-negative type strain genomes revealed that most cultured phyla (26 out of 29) have consistent predictions between SignalP-4.1 and SignalP-5.0 (1.1 ± 0.2 [S.D.] times more on average) (Supplementary Table 1). Note that the reference databases used by SignalP-4.1 and SignalP-5.0 are similar in phylogenetic composition and diversity (Supplementary Table 1). Through further analysis of genomes from 33 candidate phyla, we discover that only two candidate phyla, “*Ca*. Calescamantes” (EM3) and “*Ca*. Microgenomates” (OP11) within “*Ca*. Patescibacteria”, have RT761-like signatures (Supplementary Fig. 10) (1.8 ± 0.6 [S.D.] times more in SignalP-5.0 on average for other candidate phyla), indicating that the above features of RT761 are not an artifact from lack of *Ca*. Atribacteria-derived sequences in the SignalP reference databases. Comparison of hydrophobicity, signal peptide length, and number of NT cationic residues interestingly revealed no differences between Sec-secreted proteins predicted by SignalP-5.0 and SignalP-4.1, suggesting that the Sec-secreted proteins of RT761 may have unique signal peptides that could only be predicted through integration of a recurrent neural network implemented in SignalP-5.0. Intriguingly, we only observed RT761-like signatures (high genomic proportion of proteins with transmembrane helices and underestimation of Sec-secreted proteins by SignalP-4.1) in three other cultured phyla with unique cell structures (Fig. 3): *Thermotogae* members (outer toga^12^), *Dictyoglomi* (multicell-spanning outer envelope^21^), and *Caldiserica* (electron-lucent outer envelope^22^). In total, comparison of genomic transmembrane and extracellular protein abundance signatures may serve as a new approach for identification of bacterial lineages with unique cell membrane structure and is, thus, distinct from currently available genotype-based cell morphology prediction approaches (e.g., RodZ for rod-shape and lipid A synthesis genes for gram-negative structure).

### Syntrophic physiology

In addition to the unique cell structure and genomic features, we found that strain RT761 can benefit from syntrophic interaction with methanogenic archaea. RT761 degraded glucose, producing H_2_, acetate, CO_2_, and ethanol (trace levels) as end products and could not utilize exogenous electron acceptors for anaerobic respiration (i.e., nitrate, ferric iron, and sulfate). Although RT761 growth was inhibited by accumulation of hydrogen during cultivation with glucose, addition of a hydrogen-consuming methanogenic archaeon increased the growth rate and maximum cell density of RT761 (Supplementary Fig. 11). RT761 can theoretically shift to ethanol fermentation as an alternative electron disposal route but only generates a small amount, indicating that RT761 primarily relies on hydrogen formation to maintain cellular redox balance. Thus, in contrast to most hydrogen-producing bacteria, RT761 highly depends on syntrophic association with a hydrogen-scavenging methanogen for ideal growth. Such dependence of sugar degradation on a syntrophic partner is thought to be important in anoxic ecosystems^23,24^. Thus far, sugar-degrading organisms that benefit from syntrophic interactions have been identified in *Firmicutes* (*Syntrophococcus sucromutans*^25^, *Bacillus stamsii*^26^) and the class *Anaerolineae* of *Chloroflexi* (*Anaerolinea thermolimosa*^27^, *A. thermophila*^23^, *Bellilinea caldifistulae*^28^, *Flexilinea flocculi*^29^, and *Longilinea arvoryzae*^28^). Unlike these organisms, RT761 does not produce lactate, lacks NiFe hydrogenases, and rather possesses energy-conserving complexes often associated with nonsugar-degrading syntrophic organisms—an ion-translocating NADH:ferredoxin oxidoreductase Rnf and both a trimeric and tetrameric FeFe hydrogenase^5^ (Table 1). This suggests that the sugar-degrading strategy of RT761 differs from hitherto isolated sugar degraders that benefit from syntrophic interaction. We speculate that RT761 may avoid ethanol production as continuous exposure to acetaldehyde generated through ethanol fermentation could cumulatively damage chromosomal DNA, especially due to the slow growth rate (doubling time of 5.1 days). Similar metabolisms that are theoretically possible or thermodynamically required for association with methanogens (e.g., sugar degradation producing hydrogen and syntrophic propionate oxidation) have been predicted in cultivation-independent analyses of “*Ca*. Atribacteria”^4,5,30,31^. The observed physiology of RT761 justifies the prevalent detection of environmental clones of “*Ca*. Atribacteria” across Earth’s anoxic ecosystems favoring fermentation and syntrophy^5^.

**Table 1:** Fermentation products and genome-predicted metabolic properties of semi-syntrophic anaerobes that benefit from interaction with methanogenic archaea.

Organism	1	2	3	4	5	6	7	8
Growth Temp. (˚C)	45	50	55	55	37	37	37	30
Substrate	Glc	Glc	Glc	Suc	Glc	Suc	Frc	Glc
*Fermentation products*								
Hydrogen	++	++	++	++	++	++	++	++
Acetate	++	++	++	++	++	++	++	++
Ethanol	+/−	−	−	−	−	−	nd	−
Lactate	−	++	+	++	++	++	nd	+
Succinate	−	−	+	−	++	−	nd	+/−
Formate	−	−	+	++	++	−	nd	++
Propionate	−	−	−	+	++	−	nd	−
Pyruvate	−	−	−	+	−	−	nd	−
*Fermentation pathways*								
Pyruvate:Fd oxidoreductase	○	○	○	○	○	○		
Pyruvate-formate lyase	○	○				○		
Lactate dehydrogenase		○	○	○	○	○		
Malate fermentation		○	○	○		○		
Succinate fermentation		○		○		○		
Methylmalonyl-CoA pathway		○				○		
Propanediol fermentation		○			○			
Alcohol dehydrogenase	○	○	○	○	○	○	No genome available	No genome available
*H*_*2*_*/formate metabolism & energy conservation*								
NADH:Fd oxidoreductase (Rnf)	○							
Trimeric FeFe hydrogenase	○				○			
Tetrameric FeFe hydrogenase	○							
Hox NiFe hydrogenase			○			○		
Mbh NiFe hydrogenase		○	○	○				
Unknown NiFe hydrogenase			○			○		
FdhAB Formate dehydrogenase				○		○		
FdhA associated with HydBC				○				
FdhA Formate dehydrogenase			○					

Organisms 1, RT761(this study); 2, *Anaerolinea thermolimosa* IMO-1^27^; 3, *Anaerolinea thermophila* UNI-1^23^; 4, *Bellilinea caldifistulae* GOMI-1^28^; 5, *Flexilinea flocculi* TC1^29^; 6, *Longilinea arvoryzae* KOME-1^28^; 7, *Syntrophococcus sucromutans* S195^25^; 8, *Bacillus stamsii* BoGlc83^26^. Fermentation products: ++ = major fermentation product; + = minor fermentation product; +/− = product generated at very low concentrations or only detected under certain conditions; nd = not determine. Fermentation pathways and other genome-predicted features: ○ = detected.

*nd* not determine, *Glc* glucose, *Suc* sucrose, *Frc* fructose.

Based on unique phenotypic, genotypic and phylogenetic characteristics, including cell structure/regulation potentially more complex than a typical prokaryote, we propose strain RT761 as a new species, *Atribacter laminatus* gen. nov., sp. nov. Based on the phylogenetic and phylogenomic analyses, we further propose a new phylum *Atribacterota* phy. nov.

### Description of *Atribacter* gen. nov

*Atribacter* (A.tri.bac’ter. L. masc. adj. ater, black; N.L. masc. n. bacter, a rod; N.L. masc. n. Atribacter, a black bacterium, pertaining to the original candidate phylum name “*Ca*. Atribacteria”^4^).

Obligately anaerobic, Gram-negative, non-motile, non-spore-forming, rod cells that are tapered with pointed ends. The major end products from glucose degradation is acetate, hydrogen, and carbon dioxide. No anaerobic respiration with nitrate, sulfate, or Fe(III) is observed. The cellular fatty acids are C_15:0_ (51% of the total), C_18:0_ (21%), C_16:0_ (10%), iso-C_15:0_ (10%), iso-C_13:0_ 3OH (6%), and C_18:1_ *cis*9 (2%). DNA G+C content of the type species is 38.69 mol%. The type species is *Atribacter laminatus*.

### Description of *Atribacter laminatus* sp. nov

*Atribacter laminatus* (L. fem. n. lamina, layer; N.L. masc. adj. laminatus, layered, pertaining multilayered cell structure).

Shows the following characteristics in addition to those given for the genus. Cells are a rod or ovoid shape tapered with pointed ends with 0.6–0.8 μm wide and 1.3–1.8 μm long. Grows at 20–50 °C (optimally at 45 °C), at pH 6.4–8.2 (optimally at pH 7.3) and in the presence of 0.01–0.6 M NaCl (optimally in 0.1 M NaCl). Growth occurs with glucose, fructose, galactose, rhamnose, xylose, mannose, sucrose, cellobiose, raffinose, pectin, mannitol, and sorbitol. Ribose, arabinose, lactose, maltose, trehalose, melibiose, starch, cellulose, or gelatin are not utilized. In pure culture, the end products of glucose (1 mM) degradation are acetate (1 mM), hydrogen (1 mM), and carbon dioxide. Yeast extract is required for growth. Peptone or casamino acids did not stimulate growth. Growth is enhanced in co-culture with a hydrogen-scavenging methanogen. Sensitive to chloramphenicol, kanamycin, neomycin, rifampicin, and vancomycin, but resistant to ampicillin and neomycin. Colonies are light brown circular and convex disks on the deep agar slant. The type strain, RT761^T^ (=NBRC 112890^T^=DSM 105538^T^), was isolated from a slurry of sediments and formation water derived from a deep sedimentary, natural-gas-bearing saline aquifer in Japan.

### Description of *Atribacteraceae* fam. nov

*Atribacteraceae* (A.tri.bac.te.ra.ce’ae. N.L. masc. n. *Atribacter* type genus of the family; suff. -aceae, ending to denote a family; N.L. fem. pl. n. Atribacteraceae the family of the genus *Atribacter*).

The description is the same as for the genus *Atribacter*. Type genus is *Atribacter*.

### Description of *Atribacterales* ord. nov

*Atribacterales* (A.tri.bac.te.ra’les. N.L. masc. n. *Atribacter* type genus of the order; suff. -ales, ending to denote an order; N.L. fem. pl. n. Atribacterales the order of the genus *Atribacter*).

The description is the same as for the genus *Atribacter*. Type genus is *Atribacter*.

### Description of *Atribacteria* classis nov

*Atribacteria* (A.tri.bac.te’ri.a. N.L. masc. n. *Atribacter* type genus of the type order of the class; suff. -ia, ending to denote a class; N.L. neut. pl. n. Atribacteria the class of the order *Atribacterales*).

The description is the same as for the genus *Atribacter*. Type order is *Atribacterales*.

### Description of *Atribacterota* phyl. nov

*Atribacterota* (A.tri.bac.te.ro’ta. N.L. neut. pl. n. *Atribacteria* type class of the phylum; N.L. neut. pl. n. Atribacterota the phylum of the class *Atribacteria*).

The phylum *Atribacterota* is defined based on phylogenetic and phylogenomic analyses of the sole isolated strain RT761^T^ and uncultured representatives from various environments. Type order is *Atribacterales*.

## Methods

### Sample collection

The sediment and formation water samples were collected from a settling pond that was placed downstream of a commercial gas, and water producing well to remove suspended sand particles from the formation water in Mobara, Chiba prefecture, Japan. The samples came from the gas-bearing aquifers in the screened depth range of 490–900 m that consist of repeating sequences of turbidite (alternating beds of sandstone and mudstone) in the Otadai and Kiwada formations. These sediments were deposited in deep marine environments during the Plio-Pleistocene periods^32,33^. The water temperature was 24.4 °C, the pH was 7.7, and the redox potential was −213 mV. The Cl^−^ concentration was 17,000 mg l^−1^, and the sulfate concentration was <5 mg l^−1^ (detection threshold). The natural gases produced in this area composed mainly of methane (99%), and the origin of methane was suggested to be of biogenic based on stable isotopic analysis^34^.

The samples were collected in sterilized glass bottles with butyl rubber stoppers and screw caps. The bottle was purged with N_2_ gas prior to sample collection and was filled with the water to maintain the samples under anaerobic conditions.

### Enrichment culture and isolation

Sediment samples were mixed at a 1:2 volume ratio with a formation water to make slurry samples in an anaerobic chamber. The slurry samples were dispensed as 20 ml-aliquots into 70-ml serum vials and were then sealed using butyl rubber stoppers and aluminum crimps in an anaerobic chamber. The slurries were incubated without the addition of any nutrients under an atmosphere of N_2_/CO_2_ (80:20) at a temperature higher than the water temperatures of original environments (45 °C rather than 25 °C). After 90 days, 2 ml of the methane-producing culture of slurry sample was inoculated into a saline mineral medium^35^ supplemented with 1 g l^−1^ glucose, 1 g l^−1^ Bacto peptone (BD), 0.1 g l^−1^ yeast extracts (BD), 5 mM coenzyme M (2-mercaptoethane sulfonic acid), and 0.1 mM titanium (III) citrate (used as a reducing agent). The saline mineral medium contained 350 mM NaCl, 30 mM NaHCO_3_, 15 mM MgCl_2_⋅6H_2_O, 10 mM NH_4_Cl, 1 mM KH_2_PO_4_, 1 mM CaCl_2_⋅2H_2_O, 1 ml l^−1^ trace elements solution^35^, 1 ml l^−1^ vitamin solution^35^, and 1 ml l^−1^ resazurin solution^35^. Cultivation was performed in 75-ml serum vials containing 20 ml of medium under an atmosphere of N_2_/CO_2_ (80:20). Enrichment cultures were grown, followed by successive transfer six times at intervals of approximately 80 days. Individual cells were isolated in a pure culture using the deep agar slant method combined with dilution-to-extinction method^35^ (10-fold dilutions from 10^−1^ to 10^−8^) with a saline mineral medium supplemented with 1 g l^−1^ glucose, 0.1 g l^−1^ yeast extracts, 0.1 mM titanium (III) citrate, and 8 g l^−1^ agar. After 40 days of incubation, a single colony was picked from 10^6^-dilution culture and transferred to fresh liquid medium. This procedure was repeated three times. Purity of the culture was verified by microscopy and further confirmed by no contaminant sequences in DNA sequencing data of genomic DNA. The pure culture of strain RT761 was incubated at 45 °C in saline mineral medium amended with 16 mM glucose, 0.2 g l^−1^ yeast extracts and 0.5 g l^−1^ cysteine hydrochloride. In co-culture with methanogenic archaeon, *Methanothermobacter thermoautotrophicus* strain Delta H, 5 mM coenzyme M and 0.2 g l^−1^ Na_2_S⋅9H_2_O were also added.

### Genomic and transcriptomic analyses

Genomic DNA of strain RT761 was extracted using the Blood and Cell Culture DNA Maxi Kit (QIAGEN, Venlo, Netherlands) according to the manufacturer’s instructions. DNA was sequenced using a PacBio RS II system (PacBio, Menlo Park, CA) with one single-molecule real-time (SMRT) cells at Hokkaido System Science (Sapporo, Japan). Sequence assembly was carried out using the GS De Novo assembler Newbler (version 2.3). Gene identification and annotations were performed using annotated by Prokka v1.13^36^ and CD-Search^37^. To search for unique N-terminal extensions, all RT761 protein-coding genes were aligned with the Genbank RefSeq database^38^ using BLASTP^39^ and compared with all top 250 BLASTP hits filtered with >30% similarity and >70% coverage. Secondary structure of amino acid sequences of N-terminal extension associated with FtsZ was predicted using JPred4^40^. Transmembrane proteins and signal peptides were predicted using TMHMM v2.0^41^ (default options) and SignalP (v4.1^19^ and v5.0^20^ using the gram-negative option and default options for the remaining settings) correspondingly, and the percentages of these proteins out of the total number of ORFs were compared to those in all gram-negative type strain draft genomes available on the Joint Genome Institute Integrated Microbial Genomes and Microbiomes database^41,42^, and those in draft genomes of uncultured phyla that were confirmed to encode lipid A synthesis genes (lpxB, lpxC, or lpxD) in at least one draft genome. The 16S rRNA gene sequences were aligned against the SILVA v132 alignment using SILVA SINA Aligner v1.2.11^43^ with default settings. The phylogenetic tree was constructed using RAxML-NG^44^ using the generalized time reversible (GTR) model, 4 gamma categories, and 100 bootstrap iterations. Prediction/selection of conserved genes and tree construction was performed through PhyloPhlAn^45^ using default settings.

RNA was extracted from late exponential growth of both pure culture and co-culture with a methanogen *M. thermoautotrophicus* str. Delta H using the ISOSPIN Plant RNA kit (NIPPON GENE, Japan) according to the manufacturer’s instructions and was sequenced using an Illumina sequencer NovaSeq 600 system (illumina, USA) at Filgen, Inc. (Nagoya, Japan). Total RNA was depleted of ribosomal RNA via Ribo-Zero rRNA removal kit (illumina). The sequenced RNA was trimmed via Trimmomatic v0.33^46^ and mapped to the assembled genome through BBmap v37.10 (https://sourceforge.net/projects/bbmap/) to calculate the gene expression levels, which were represented by Reads Per Kilobase of transcript per Million mapped reads.

### Microscopic analyses

Cell morphology and structure was observed via phase-contrast and fluorescence microscopy (BX51; Olympus, Japan), confocal laser scanning microscopy (LSM800; ZEISS, Germany), scanning electron microscopy (SEM) (S-4500; Hitachi, Japan), transmission electron microscopy (TEM) (H-7600; Hitachi, Japan) and cryo-electron microscopy (CRYO ARM 300; JEOL, Japan). The cells in exponential phase of growth in pure culture condition were used for all microscopic observation.

Cells were washed with phosphate-buffered saline (PBS) before staining. A Gram-staining kit (BD) was used for Gram staining. Membranes of RT761 cells were strained with FM4-64 (ThermoFisher Scientific, USA) at a final concentration of 40 µg ml^−1^. DNA was strained with Hoechst 33342 (ThermoFisher Scientific) at final concentrations of 2 µg ml^−1^. RNA was strained with SYTO RNAselect (ThermoFisher Scientific) at a final concentration of 10 µM. The stained sample was incubated for 1 h at 30 °C and observed under confocal laser scanning microscope.

For fluorescence in situ hybridization, the cells were fixed in 1% paraformaldehyde at 4 °C for overnight and stored in 99% ethanol-PBS (1:1) at −20 °C. The fixed cells were incubated in a moisture chamber with a hybridization buffer (0.9 M NaCl, 0.01% sodium dodecyl sulfate, 20 mM Tris-HCl, pH 7.2 containing fluorescently labeled probes (0.5 pmol µl^−1^). After incubation at 46 °C for 2.5 h, the buffer was replaced with washing solution (0.9 M NaCl, 0.01% sodium dodecyl sulfate, 20 mM Tris-HCl, pH 7.2). The sample was incubated at 48 °C for 30 min and observed under a fluorescence phase-contrast microscope. An oligonucleotide probe targeting the 16S rRNA gene was Cy-3-labeled EUB338 probe (5′- GCTGCCTCCCGTAGGAGT-3′).

For SEM observation, the cells were fixed with 2% glutaraldehyde in 0.1 M sodium phosphate buffer (pH7.2) at 4 °C for 2 h, postfixed with 1% osmium tetroxide at room temperature for 1 h, dehydrated through a graded ethanol series followed by 3-methylbutyl acetate for 20 min, dried with a critical point dryer (JCPD-5; JEOL), and finally coated with gold.

For cryo-electron microscopy and tomography, 2 μl of the cell culture were placed on glow-discharged holey carbon grid (Quantifoil R 1/4 Cu grid, Quantifoil MicroTools GmbH, Germany), and the grid was automatically blotted at 22 °C and 80% humidity and plunged into liquid ethane using a Leica EM GP (Leica Microsystems, Austria). The frozen grid was mounted onto a liquid-nitrogen cryo-specimen holder and loaded into a CRYO ARM 300 equipped with a cold-field emission electron gun operating at 300 kV, a hole-free phase plate^47^ and an omega-type in-column energy filter with an energy slit width of 30 eV. The images were recorded on K3 direct detection camera (GATAN, USA) at a nominal magnification of 10,000–15,000× (corresponding to an imaging resolution of 3.3–4.9 Å per pixel, with the total dose under 1.5 electrons per Å^2^ using a low dose system). For observation of two leaflets of cellular layers, the images were recorded at a magnification of 60,000× (corresponding to an imaging resolution of 0.8 Å per pixel, with the total dose under 25 electrons per Å^2^ using a low dose system). Tilt series images were collected automatically in a range of ±60° at 2° increments using the SerialEM v3.8.0 beta (http://bio3d.colorado.edu/SerialEM)^48^. The total electron dose on the specimen per tilt series was kept under approximately 90 electrons per Å^2^ to minimize radiation damage. The tilt series were aligned using gold fiducials, and tomograms were reconstructed using the IMOD v4.9.12^49^. The 3D segmentation of eight times binned volumes including surface rendering and smoothing to generate the final tomographic model was performed with Amira v6.3.0 (ThermoFisher Scientific) according to the previously described method^50^. In brief, segmentation of each structure was traced manually using brush tools in Amira. The thickness of layers, distances between each layer and size of ribosomes were determined from 16 tomographic slice pictures of four different cells. The percentage of jagged structure in outline of outermost layer was measured from 15 cells of two-dimensional tomographic projection images.

For TEM observation, the cells were fixed with 2.5% glutaraldehyde in 0.1 M sodium cacodylate buffer (pH7.4) at 4 °C for 3 h and then postfixed with 1% osmium tetroxide at 4 °C for 90 min. The fixed cells were suspended in 1% aqueous uranyl acetate at room temperature for 1 h. The suspended cells were embedded in 1.5% agarose and dehydrated through a graded ethanol series. The dehydrated blocks were embedded in Epon812 resin. Ultrathin sections were cut with an ultramicrotome (Leica EM UC7), mounted on copper grids, and stained with uranyl acetate and lead citrate.

### Physiological characterization

All physiological experiments were performed in triplicate. The effects of temperature, pH, and concentrations of NaCl on cell growth, utilization of carbohydrates and sensitivity to antibiotics were determined by hydrogen production in gas phase of cultures. Hydrogen and methane in gas phase of cultures were measured with a gas chromatography equipped with a thermal conductivity detector (GC-8A; Shimadzu, Japan). Glucose, acetate, and ethanol in liquid phase of cultures were measured with a high-performance liquid chromatography (HPLC) (LC20; Shimadzu) with Shim-pack SPR-H column (Shimadzu) or HPLC (LC-2000Plus, Jasco) with Aminex HPX-87H column (BIO-RAD). Methyl esters of cellular fatty acids were identified and quantified via a gas chromatography-mass spectrometry (M7200A GC/3DQMS system; Hitachi).

### Quantitative PCR

SYBR green-based real-time PCR was run on a CFX Connect real-time PCR detection system (Bio-Rad Laboratories Inc., USA) using the PowerUp SYBR green master mix (Applied Biosystems, USA) to quantify the population of RT761 cells. The forward and reverse primers, rt1F (5′-GCTAATACCCCATATGCTCCCTG-3′) and rt1R (5′-ACCTCGCCAACCAGCTGATGGGG-3′), were designed from the 16S rRNA gene sequences of strain RT761. The length of amplified products was 62 bp. Total DNA was extracted from pure- or co-cultures using an ISOSPIN Fecal DNA (NIPPON GENE). Standard curves for quantification were determined based on 10-fold serial dilutions of the target PCR products of strain RT761 at known concentrations. All reactions, including the non-template control, were performed in triplicate. The presence of a single PCR product without any nonspecific amplicons was confirmed via agarose gel and melting curve analyses. The PCR product was sequenced by Sanger sequencing to confirm the amplification of 16S rRNA gene from strain RT761. All qPCR runs showed no PCR amplifications from non-template control and culture samples without adding RT761 cells, and had efficiency levels of approximately 95%, with an *R*^2^ of >0.99. Cell growth rate was estimated using 16S rRNA gene copy number as a proxy for cell population.

### Statistics and reproducibility

For phase-contrast microscopic observation, a representative section of one field of view (Fig. 1a) was selected from ten fields of view using cells from three independent cultures. For fluorescence microscopic observation, a representative section of one field of view (Fig. 2) was selected from eight fields of view from two independent experiments. For FISH observation, a representative section of one field of view (Supplementary Fig. 6a) was selected from four fields of view from two independent experiments. For SEM observation, a representative section of one field of view (Fig. 1b) was selected from 17 fields of view using cells from three independent cultures. For TEM observation, a representative section of one field of view (Fig. 1f) was selected from 29 fields of view using cells from three independent cultures. For cryo-electron microscopy (at high electron dose), a representative section of one field of view (Supplementary Fig. 3) was selected from four fields of view from two independent cultures. For cryo-electron microscopy (at low electron dose), a representative section of one field of view (Supplementary Fig. 7) was selected from 30 fields of view using cells from two independent cultures. For CET, four different cells from two independent cultures were selected for tomographic analysis, and a representative section of one slice image with different binned volumes (Fig. 1c, d and Supplementary Figs. 4, 5, 7) from original 4092 tilt series in each dataset. Among these four cells, one cell was selected for segmentation and 3D reconstruction (Fig. 1e).

### Reporting summary

Further information on research design is available in the Nature Research Reporting Summary linked to this article.

## Supplementary information

Supplementary Information

Peer Review File

Description of Additional Supplementary Files

Supplementary Data 1

Supplementary Data 2

Supplementary Data 3

Supplementary Movie 1

Supplementary Movie 2

Supplementary Movie 3

Supplementary Movie 4

Reporting Summary

## Data Availability

The draft genome sequences and annotation data of strain RT761 are available in NBCI BioProject under accession number PRJNA528842. The type strain of *Atribacter laminatus*, RT761^T^ (=NBRC 112890^T^=DSM 105538^T^), has been deposited in two culture collections: NBRC (Japan) and DSMZ (Germany). All micrographic data are available at BioStudies under accession code S-BSST519. The reference genome sequences used in this study are available on the Joint Genome Institute Integrated Microbial Genomes and Microbiomes database (https://img.jgi.doe.gov/). All other data supporting the findings of this study are available within the article and its Supplementary Information. Source data are provided with this paper.

## Source data

Source Data
