# Supplementary material for: Isolation of a member of the candidate phylum ‘Atribacteria’ reveals a unique cell membrane structure
Source: Nat Commun. 2020 Dec 14;11:6381. doi: 10.1038/s41467-020-20149-5 (PMC7736352; doi:10.1038/s41467-020-20149-5)
Supplement: Supplementary file 1 — Supplementary Information [file 41467_2020_20149_MOESM1_ESM.pdf]

Supplementary information for

Isolation of a member of the candidate phylum 'Atribacteria' reveals a unique cell membrane structure

Taiki Katayama, Masaru K. Nobu, Hiroyuki Kusada, Xian-Ying Meng, Naoki Hosogi, Katsuyuki Uematsu, Hideyoshi Yoshioka, Yoichi Kamagata* and Hideyuki Tamaki*

*Corresponding authors.
Email: y.kamagata@aist.go.jp (Y.K.); tamaki-hideyuki@aist.go.jp (H.T.)

This file includes:
Supplementary Figs. 1 to 11
Supplementary Table 1

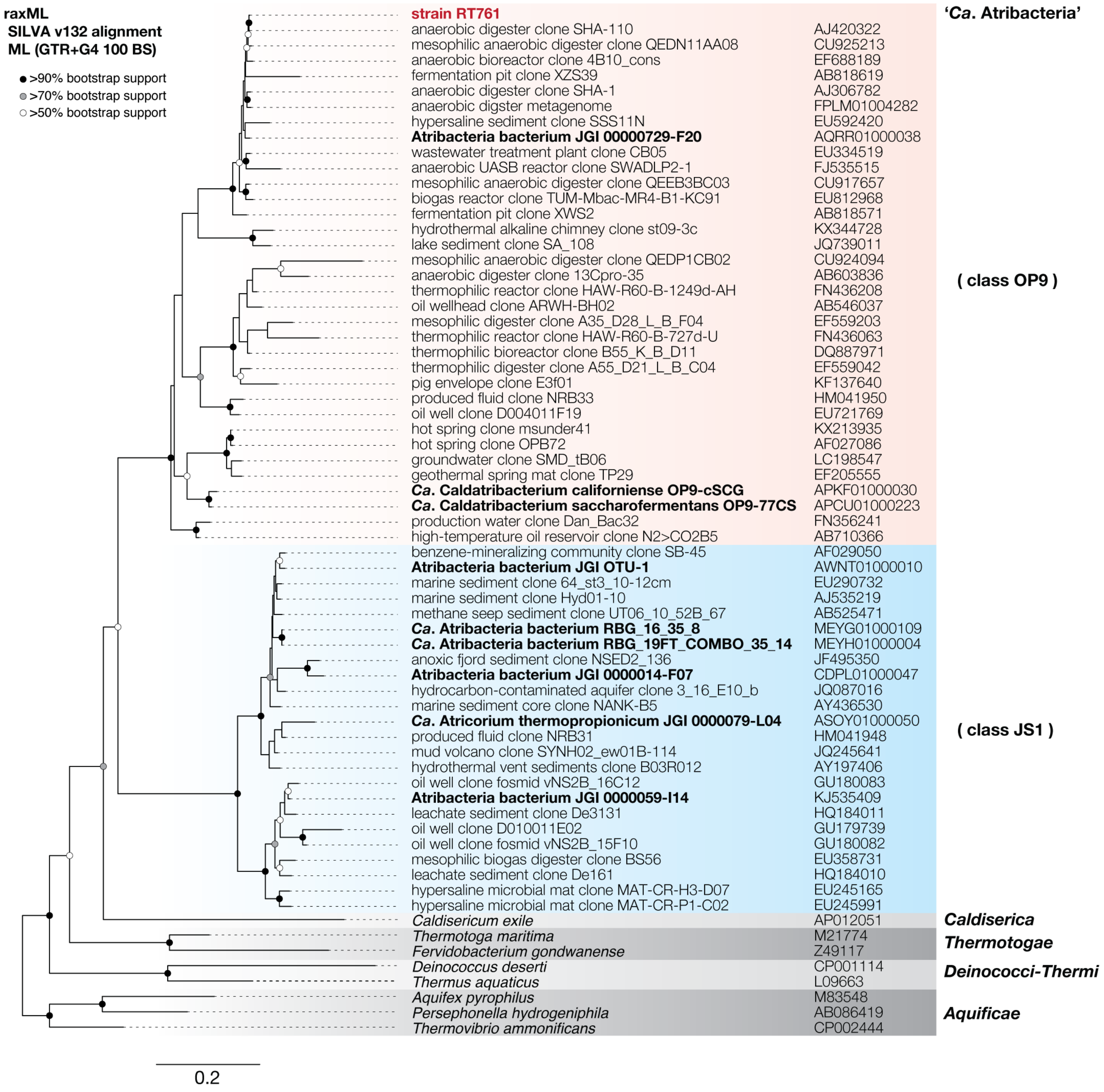

Supplementary Fig. 1. A 16S rRNA gene phylogenetic tree (Maximum likelihood tree) showing the relationship between strain RT761 (red) and relatives including single-cell genomes and metagenomic bins (bold) assigned to '*Ca*. Atribacteria' classes OP9 (red background) and JS1 (blue background). Bootstrap values greater than 50% (white circle), 70% (gray), and 90% (black) are shown.

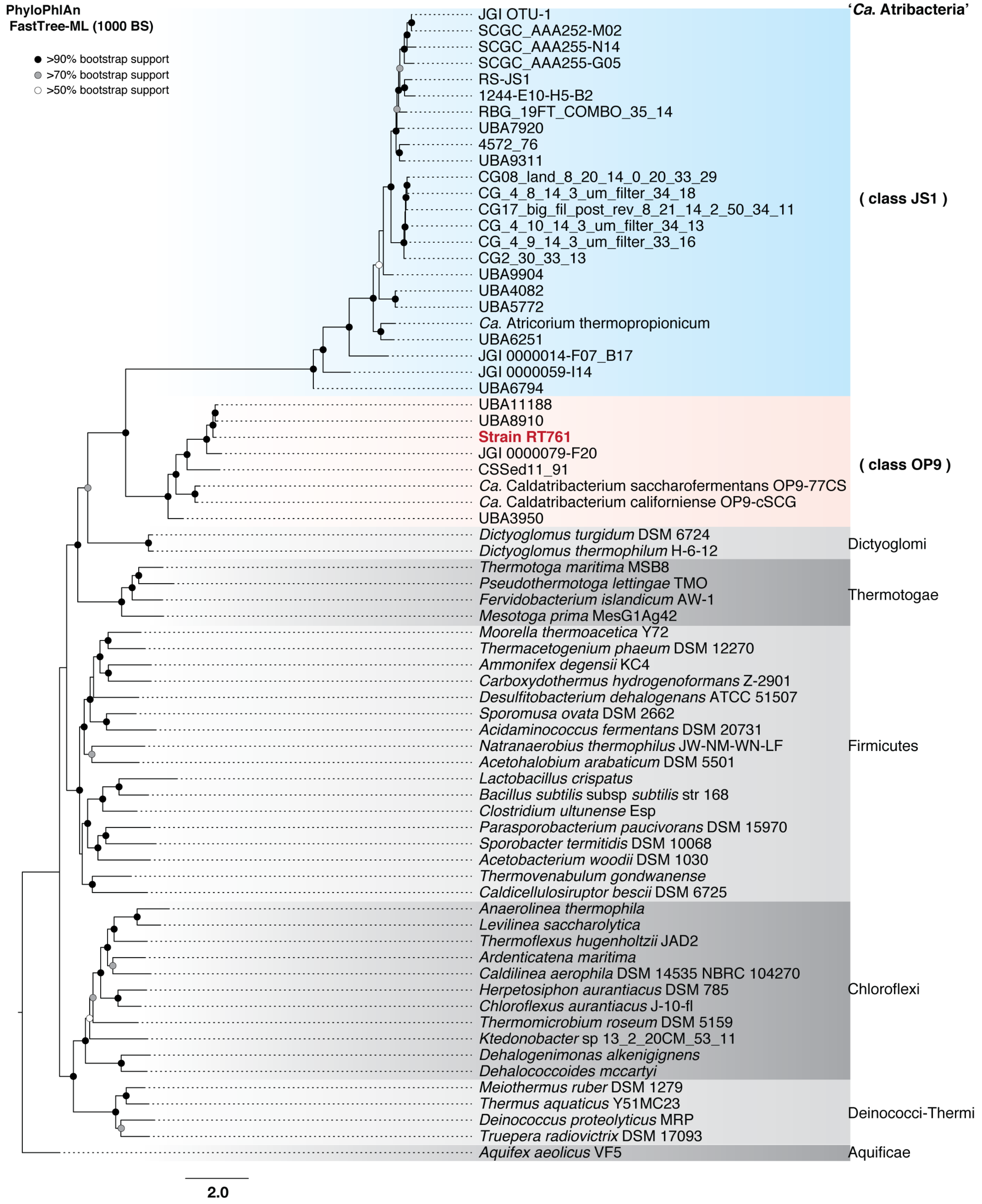

Supplementary Fig. 2. Phylogenomic analysis of strain RT761 (red) and relatives using a concatenated alignment of conserved marker genes (PhyloPhlAn). Genomes were selected for '*Ca.* Atribacteria' classes OP9 (red background) and JS1 (blue background) and other related phyla. Bootstrap values greater than 50% (white circle), 70% (gray), and 90% (black) are shown.

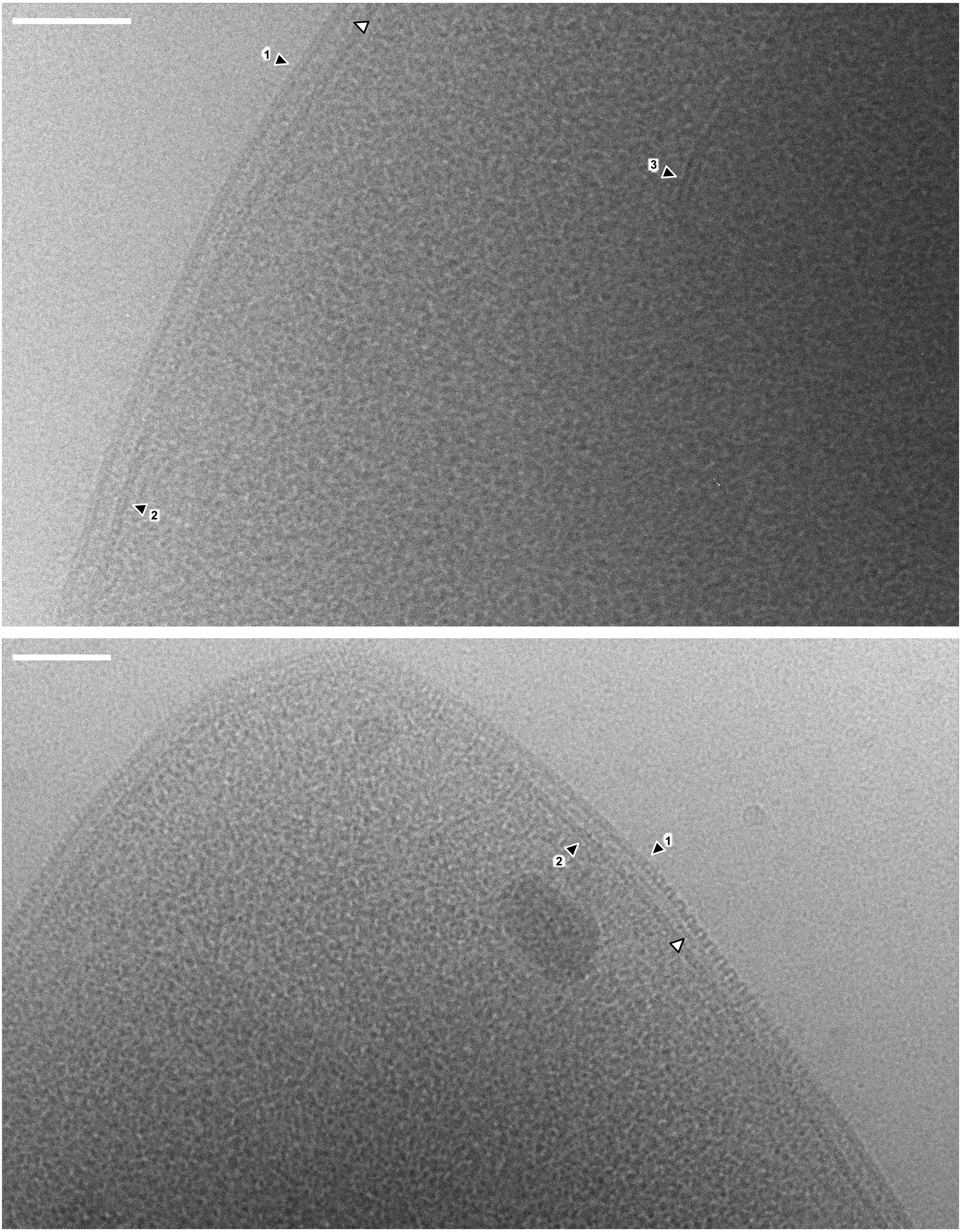

Supplementary Fig. 3. Projection electron micrographs of RT761 cell showing two leaflets of three layers [black arrowhead, outer lipid membrane-like layer (LML) (1), middle LML (2), inner LML (3)] and 2.2 nm thick layer (white arrowhead). (Scale bars: 0.05 μm)

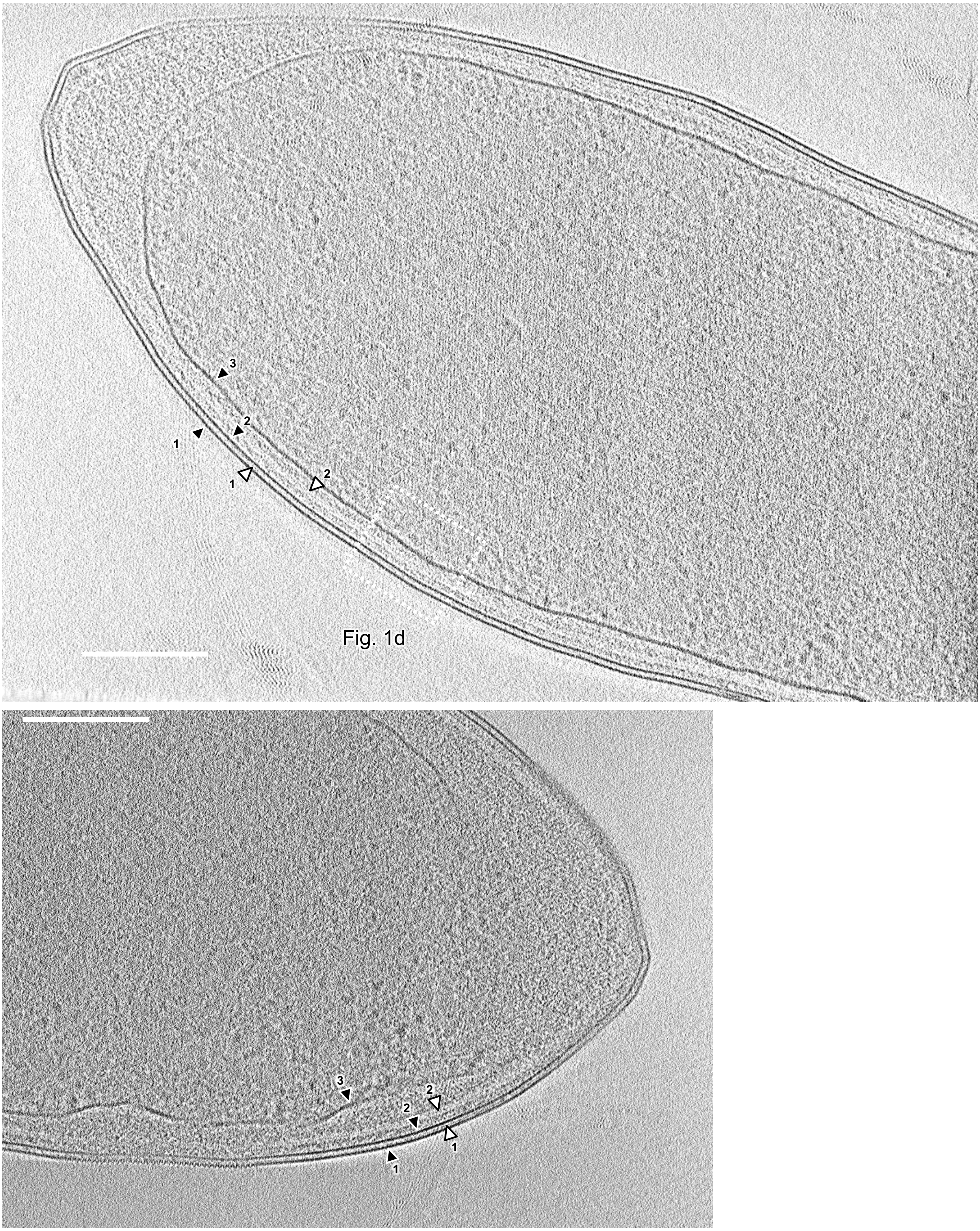

Supplementary Fig. 4. Cryo-electron tomography of RT761 cell. Enlarged view of the boxed regions are shown in Fig. 1d [for jagged structure in outer membrane (lower panel), also see Supplementary Video 4]. Black arrowheads indicate outer lipid membrane-like layer (LML) (1), middle LML (2) and inner LML (3). White arrowheads indicate 2.2 nm thick layer (1) and slant layers (2). (Scale bars: 0.2 μm)

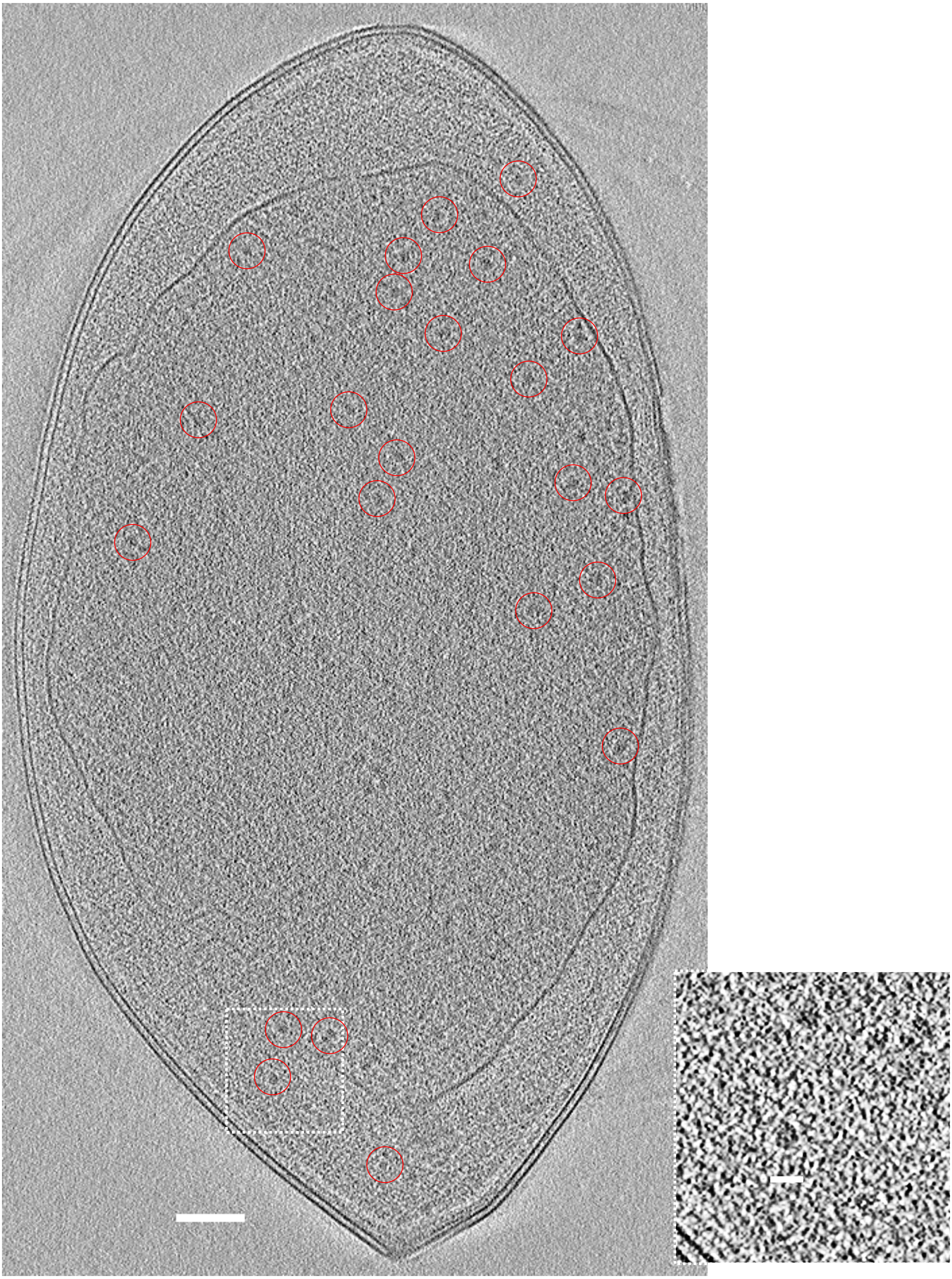

Supplementary Fig. 5. Cryo-electron tomography of RT761 cell showing the presence of particles identical to ribosome size (c.a. 20 nm) (enclosed by red circle) in both outside and inside of the inner lipid membrane-like layer. [Scale bars: 0.1 μm (20 nm in box)]

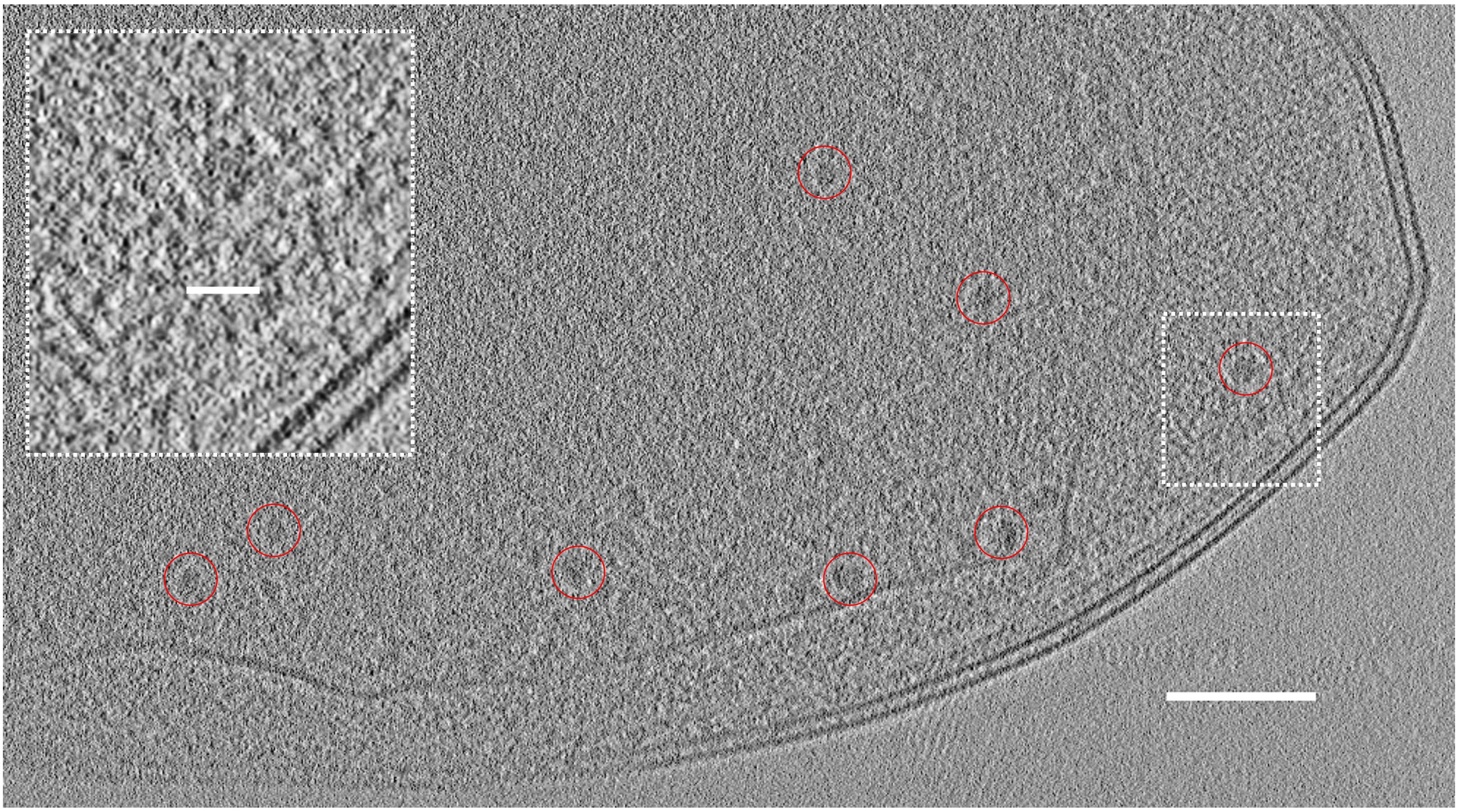

Supplementary Fig. 5. (continued) Cryo-electron tomography of RT761 cell showing the presence of particles identical to ribosome size (c.a. 20 nm) (enclosed by red circle) in both outside and inside of the inner lipid membrane-like layer. [Scale bars: 0.1 μm (20 nm in box)]

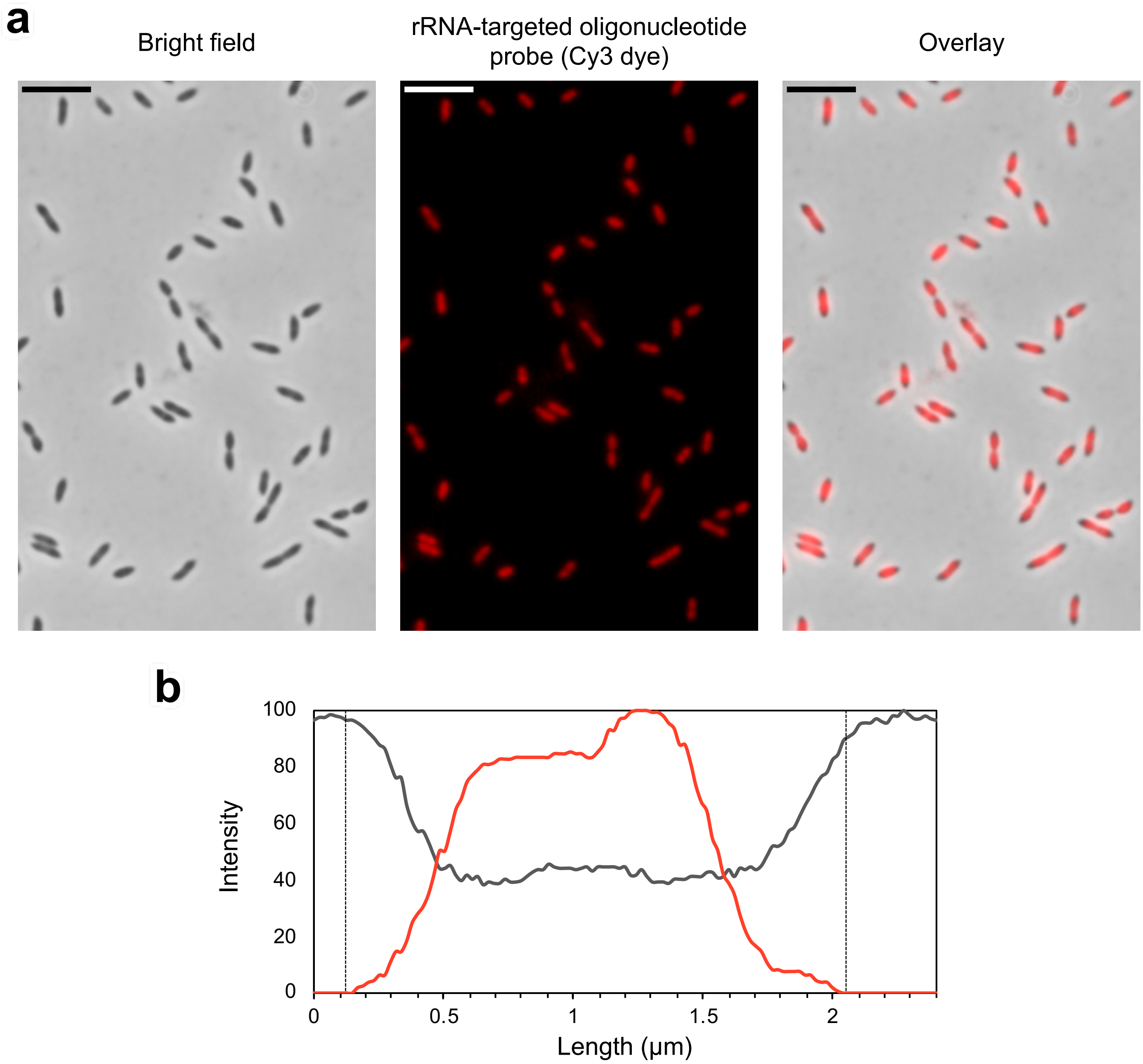

Supplementary Fig. 6. 16S ribosomal RNA (rRNA) staining of formamide-fixed cells using fluorescence in situ hybridization showing the rough distribution of ribosomes in RT761 cells. Weak fluorescence signal of stained rRNA was observed in cell poles, which entirely coincided with the cytoplasmic membrane-bounded space (CBS) (Fig. 1). (**a**) Phase contrast micrographs. (**b**) Line profiles of signal intensity of cell (black) and rRNA (red). Broken lines indicate the edges of cell observed in bright field. Source data are provided as a Source Data file. (Scale bars: 5 μm)

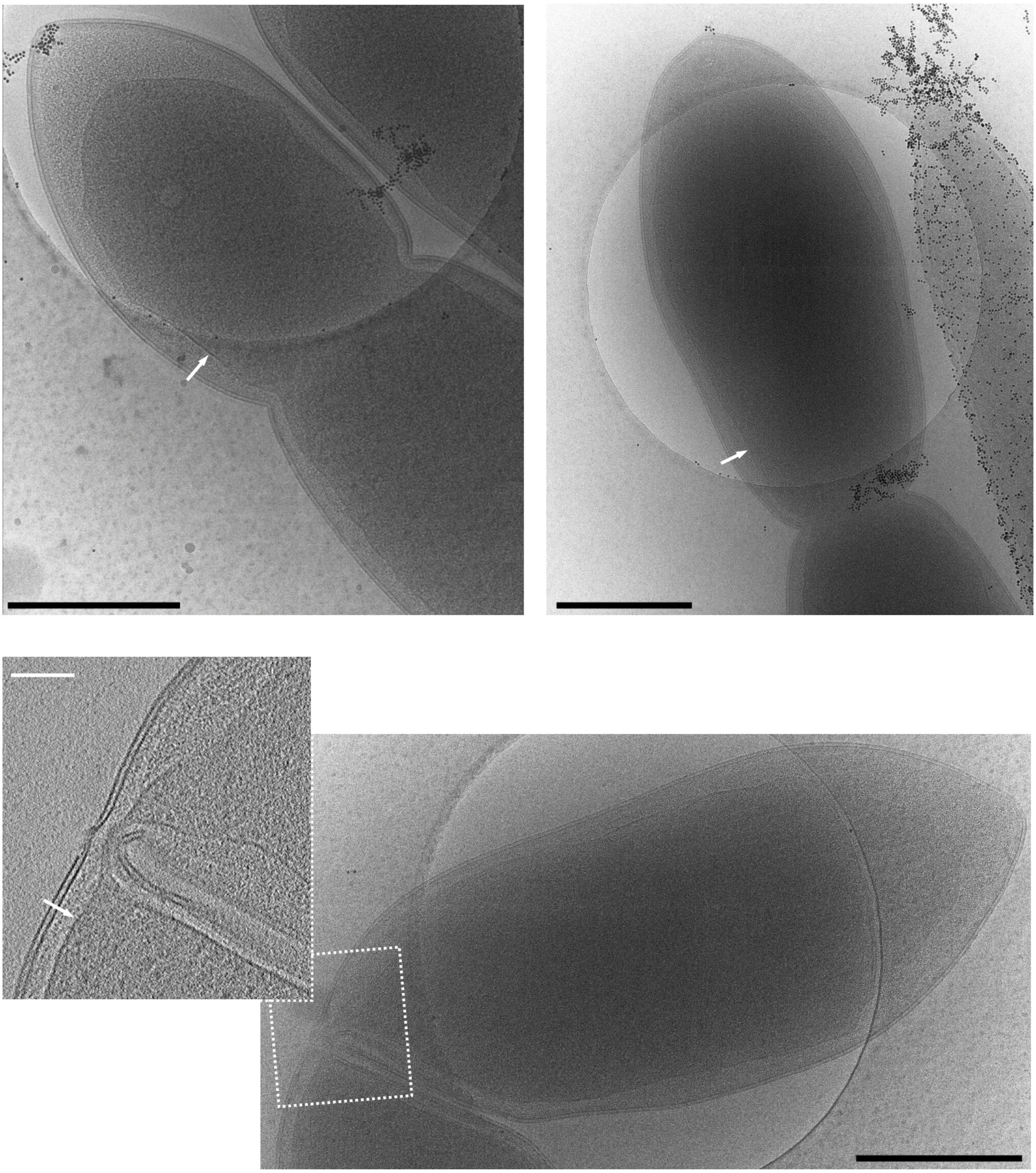

Supplementary Fig. 7. Low-dose, energy-filtered, projection electron micrographs of RT761 cell showing the invagination of membranes related to binary fission. Arrows indicate the intracytoplasmic membrane. Enlarged view of the boxed region is shown by tomographic slice (also see Supplementary Video 2). (Black bars: 0.5 μm, white bar: 0.1 μm)

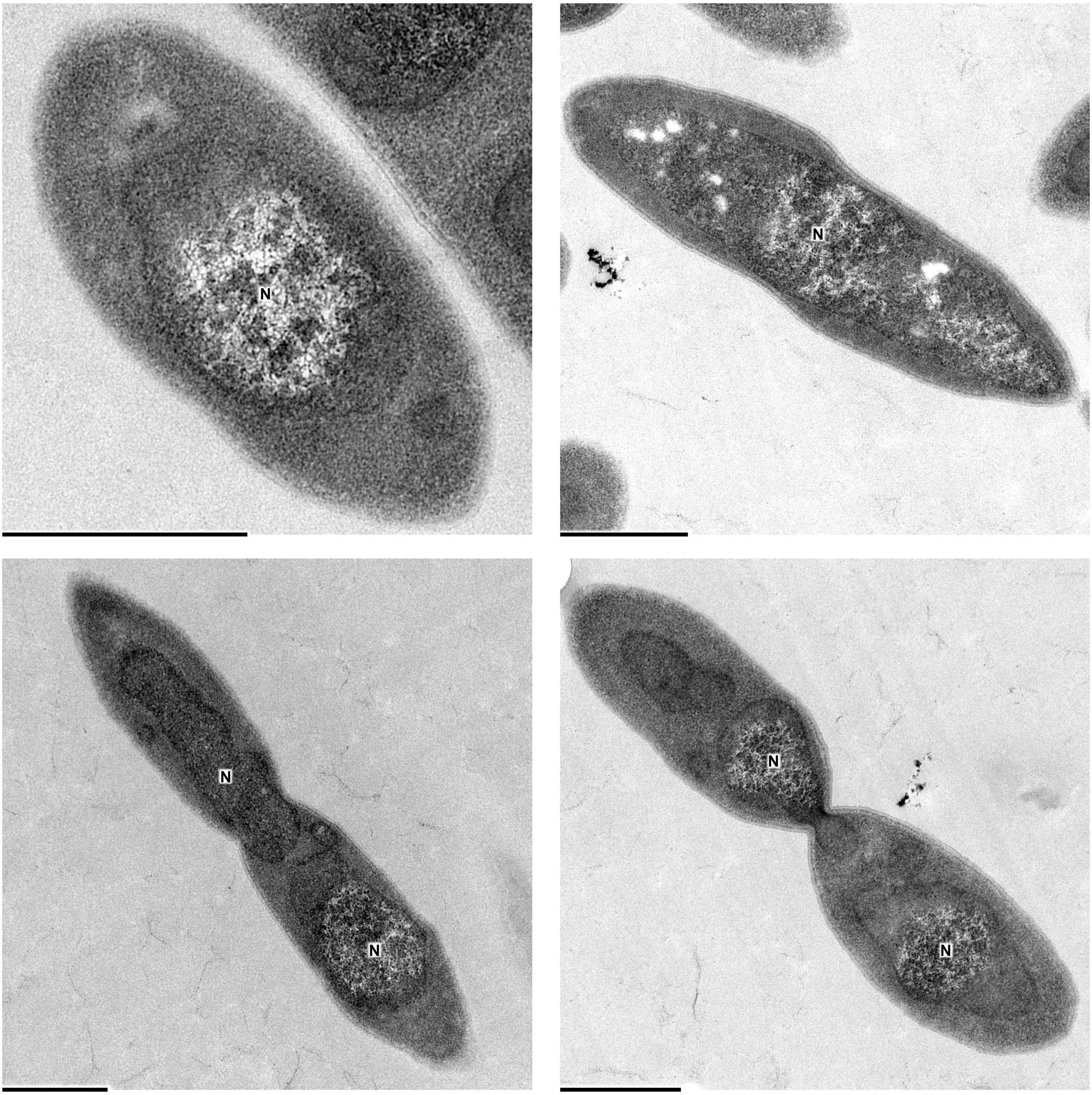

Supplementary Fig. 8. The appearance of intracytoplasmic membrane during cell division in transmission electron micrographs. Abbreviation: N, nucleoid. (Scale bars: 0.5 µm)

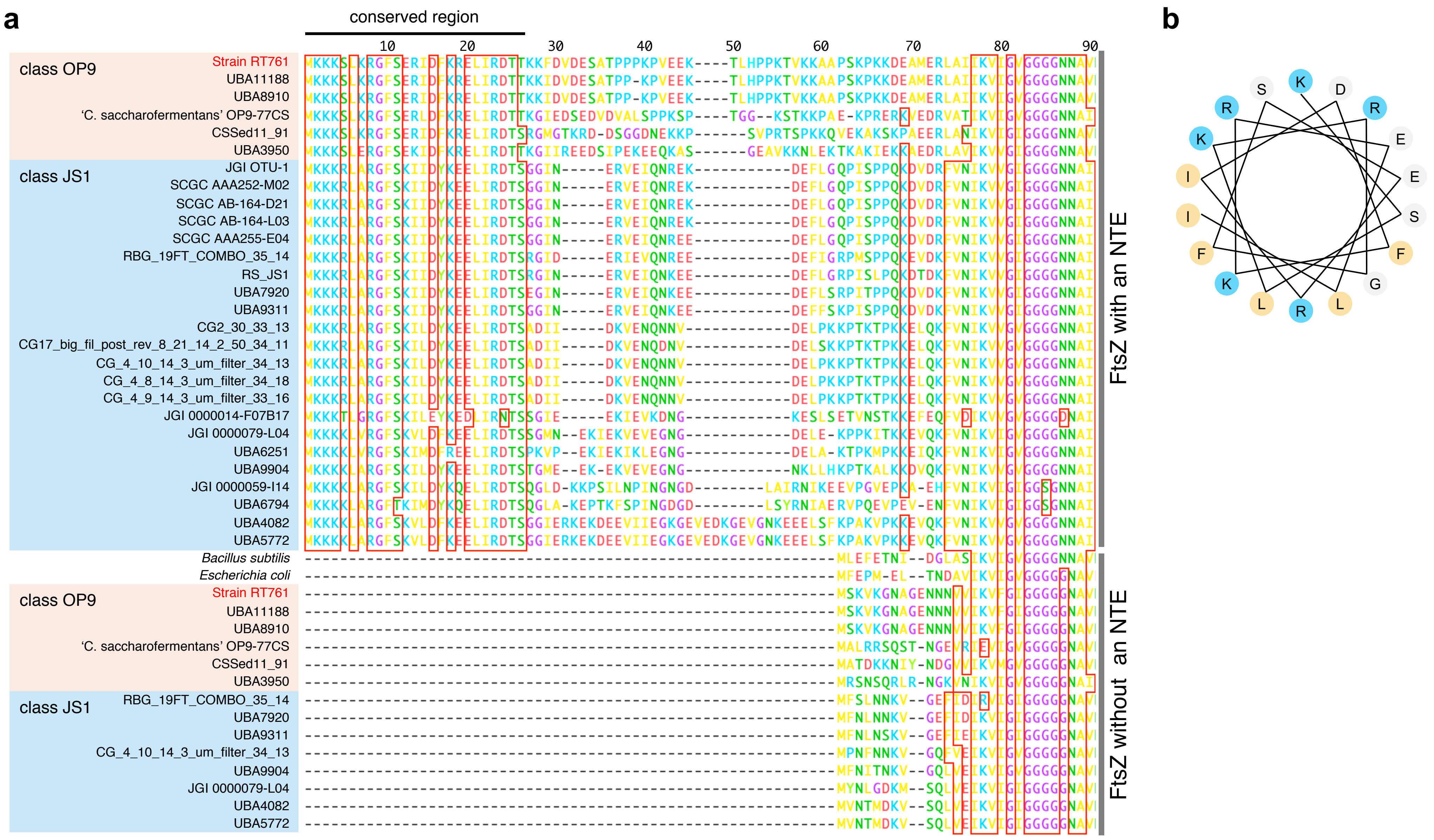

Supplementary Fig. 9. The N-terminal extensions (NTEs) of FtsZ in '*Ca*. Atribacteria' containing a conserved region that has the capacity to form an amphipathic helix. (**a**) Comparison of the *N*-terminal amino acid sequences of FtsZ with and without an NTE from '*Ca*. Atribacteria' genomes. In RT761, two FtsZ proteins shared 86% amino acid sequence similarity. (**b**) Helical wheel diagram of the conserved region from FtsZ with an NTE of RT761. Large hydrophobic and basic residues are highlighted in yellow and blue respectively.

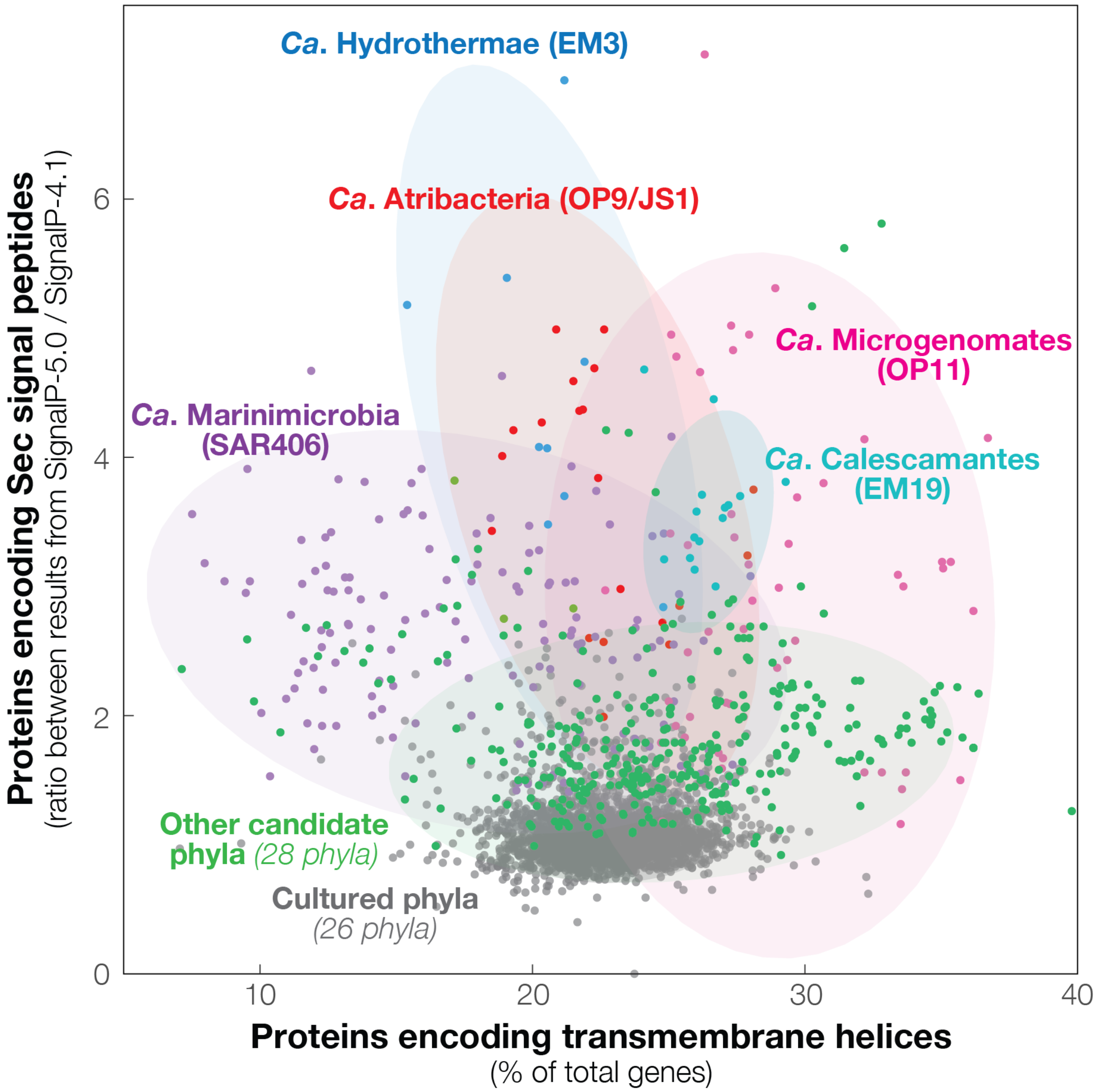

Supplementary Fig. 10. Genomic compositions of membrane-related features in yet-to-be-cultured candidate phyla. The horizontal axis shows the genomic proportion proteins encoding transmembrane helices. The vertical axis shows the ratio of proportions of proteins encoding Sec signal peptides estimated by SignalP-5.0 and SignalP-4.1. Genomes of '*Ca.* Atribacteria' (OP9 and JS1; red), '*Ca.* Microgenomates' (OP11; pink), '*Ca.* Calescamantes' (EM19; light blue), '*Ca.* Hydrothermae' (EM3; blue), '*Ca.* Marinimicrobia' (SAR406; purple), 28 other candidate phyla (green), and 26 cultured phyla (gray, see Supplementary Fig. 8) are plotted. Confidence ellipses (95%) are shown for each aforementioned group. Source data are provided as a Source Data file.

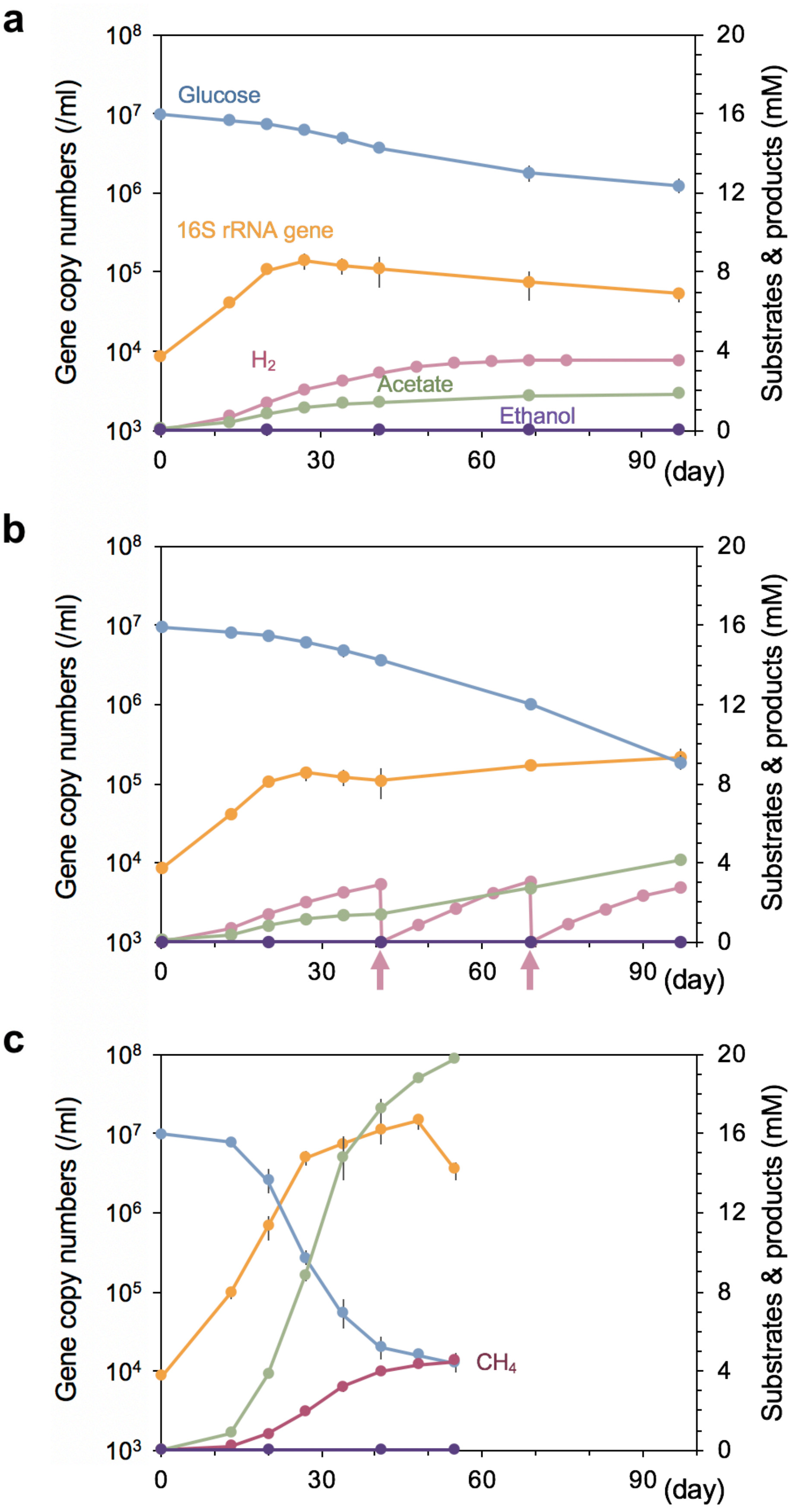

Supplementary Fig. 11. Growth and metabolic behavior of RT761 showing the effects of H_2 removal on growth. (**a**) Pure culture. (**b**) Pure culture with periodic purging of culture vial head space with N_2/CO_2 (arrow). (**c**) Co-culture with H_2-scavenging methanogenic archaeon, *Methanothermobacter thermoautotrophicus* str. ΔH. Means and standard deviation (error bars) of triplicate cultures are shown. In all culture conditions, ethanol was detected in HPLC analysis, but its concentration was too low (at least lower than 0.2 mM) for determining accurate concentrations. Source data are provided as a Source Data file.

Supplementary Table 1. Number of UniProt-reviewed proteins with signal peptide (for each phylum) included in reference database used by different SignalP versions.

Phylum	SignalP-4.1 db	SignalP-5.0 db
Firmicutes	1438	1534
Proteobacteria	5475	5955
Actinobacteria	308	605
Cyanobacteria	192	201
Proteobacteria.	9	9
Chlamydiae	125	136
Spirochaetes	134	149
Deinococcus-Thermus	12	17
Tenericutes	98	102
Fibrobacteres	3	3
Aquificae	31	33
Bacteroidetes	60	134
Chloroflexi	3	5
Thermotogae	14	20
Dictyoglomi	1	1
Chlorobi	11	14
Verrucomicrobia	2	2
Acidobacteria	1	1
Nitrospirae	1	1
Fusobacteria	0	1
Planctomycetes	0	3
