# Supplementary material for: Isolation of a member of the candidate phylum ‘Atribacteria’ reveals a unique cell membrane structure
Source: Nat Commun. 2020 Dec 14;11:6381. doi: 10.1038/s41467-020-20149-5 (PMC7736352; doi:10.1038/s41467-020-20149-5)
Supplement: Supplementary file 3 — Description of Additional Supplementary Files [file 41467_2020_20149_MOESM3_ESM.pdf]

Description of Additional Supplementary Files

File Name: Supplementary Data 1
Description: RT761 genes that have unique N-terminal sequences (≥10aa) compared with top 250 blastp hits to the Genbank RefSeq database. *Criteria 1: ≥30% similarity and ≥70% sequence overlap bidirectionally. †Criteria 2: criteria (1) & alignment start position ≥10 aa in query gene and ≤10 in target gene & query gene start position ≥10 aa and ≤100 aa later than target gene. ‡Criteria 3: criteria (1) & alignment start positions ≤9 aa for both query and target gene. Conserved domains identified through CD-Search are also shown.

File Name: Supplementary Data 2
Description: Genes of representative '*Ca*. Atribacteria' that have unique N-terminal sequences (≥10aa) compared with top 250 blastp hits to the Genbank RefSeq database. *Criteria 1: ≥30% similarity and ≥70% sequence overlap bidirectionally. †Criteria 2: criteria (1) & alignment start position ≥10 aa in query gene and ≤10 in target gene & query gene start position ≥10 aa and ≤100 aa later than target gene. ‡Criteria 3: criteria (1) & alignment start positions ≤9 aa for both query and target gene. Conserved domains identified through CD-Search are also shown.

File Name: Supplementary Data 3
Description: Expression levels of RT761 genes in two replicates (reads per kilobase of transcript per million mapped reads - RPKM) calculated using BBmap. The RPKM values were further normalized to the median expression level of all genes with mapped transcripts.

File Name: Supplementary Movie 1
Description: Cryo-electron tomographic slices of RT761 cells shown in Figure 1d

File Name: Supplementary Movie 2
Description: Cryo-electron tomographic slices of dividing cells

File Name: Supplementary Movie 3
Description: Animation of the 3D reconstruction shown in Figure 1e

File Name: Supplementary Movie 4
Description: Cryo-electron tomographic slices of jagged structures appeared in outermost lipid bilayer-like layer.
