# Supplementary material for: Isolation of a member of the candidate phylum ‘Atribacteria’ reveals a unique cell membrane structure
Source: Nat Commun. 2020 Dec 14;11:6381. doi: 10.1038/s41467-020-20149-5 (PMC7736352; doi:10.1038/s41467-020-20149-5)
Supplement: Supplementary file 11 — Reporting Summary [file 41467_2020_20149_MOESM11_ESM.pdf]

nature research

Corresponding author(s): Yoichi Kamagata and Hideyuki Tamaki

Last updated by author(s): Oct 2, 2020

Reporting Summary

Nature Research wishes to improve the reproducibility of the work that we publish. This form provides structure for consistency and transparency in reporting. For further information on Nature Research policies, see our Editorial Policies and the Editorial Policy Checklist.

Statistics

For all statistical analyses, confirm that the following items are present in the figure legend, table legend, main text, or Methods section.

n/a	Confirmed	
☐	☒	The exact sample size (n) for each experimental group/condition, given as a discrete number and unit of measurement
☐	☒	A statement on whether measurements were taken from distinct samples or whether the same sample was measured repeatedly
☒	☐	The statistical test(s) used AND whether they are one- or two-sided<br>*Only common tests should be described solely by name; describe more complex techniques in the Methods section.*
☒	☐	A description of all covariates tested
☒	☐	A description of any assumptions or corrections, such as tests of normality and adjustment for multiple comparisons
☐	☒	A full description of the statistical parameters including central tendency (e.g. means) or other basic estimates (e.g. regression coefficient) AND variation (e.g. standard deviation) or associated estimates of uncertainty (e.g. confidence intervals)
☒	☐	For null hypothesis testing, the test statistic (e.g. F, t, r) with confidence intervals, effect sizes, degrees of freedom and P value noted<br>*Give P values as exact values whenever suitable.*
☒	☐	For Bayesian analysis, information on the choice of priors and Markov chain Monte Carlo settings
☒	☐	For hierarchical and complex designs, identification of the appropriate level for tests and full reporting of outcomes
☒	☐	Estimates of effect sizes (e.g. Cohen's d, Pearson's r), indicating how they were calculated

Our web collection on statistics for biologists contains articles on many of the points above.

Software and code

Policy information about availability of computer code

Data collection	No software was used for data collection
Data analysis	Newbler v2.3 for genome sequence assemble; Prokka v1.13 and CD-search for annotation; BlastP for amino acid sequence alignment; JPred4 for prediction of secondary structure of amino acid sequence; TMHMM v2.0 for transmembrane helix prediction; SignalP v4.1 and 5.0 for signal peptide prediction; SILVA SINA Aligner v1.2.11 for 16S rRNA gene sequence alignment; Phylophlan v1 for genome tree construction; RAxML-ng v0.9.0 for maximum likelihood tree construction; Trimmomatic v0.33 for sequence trimming; BBmap v37.10 for read mapping; SerialEM v3.8.0 beta for collection of tilt series for cryo-electron tomography; iMOD v4.9.12 for reconstruction of cryo-electron tomography; and Amira v6.3.0 for 3D segmentation.

For manuscripts utilizing custom algorithms or software that are central to the research but not yet described in published literature, software must be made available to editors and reviewers. We strongly encourage code deposition in a community repository (e.g. GitHub). See the Nature Research guidelines for submitting code & software for further information.

Data

Policy information about availability of data

All manuscripts must include a data availability statement. This statement should provide the following information, where applicable:

- Accession codes, unique identifiers, or web links for publicly available datasets
- A list of figures that have associated raw data
- A description of any restrictions on data availability

The draft genome sequences and annotation data of strain RT761 are available in NBCI BioProject under accession number PRJNA528842 (https://www.ncbi.nlm.nih.gov/bioproject/PRJNA528842). The type strain of Atribacter laminatus, RT761T (= NBRC 112890T = DSM 105538T), was deposited in culture collections NBRC, Japan and DSMZ, Germany. The electron micrographs, including raw tilt-series of CET, are available at the EMPIAR (accession code

EMPIAR-10534) (https://www.ebi.ac.uk/biostudies/BioImages/studies/EMPIAR-10534). The other micrographs are available at BioStudies (accession code S-BSST519) (https://www.ebi.ac.uk/biostudies/BioImages/studies/S-BSST519). The source data underlying Figures 2b and 3 and Supplementary Figures 5b, 10 and 11 are provided as a Source Data file. The reference genome sequences used in this study are available on the Joint Genome Institute Integrated Microbial Genomes and Microbiomes database (https://img.jgi.doe.gov/). All other data supporting the findings of this study are available within the article and its Supplementary Information.

Field-specific reporting

Please select the one below that is the best fit for your research. If you are not sure, read the appropriate sections before making your selection.

☒ Life sciences ☐ Behavioural & social sciences ☐ Ecological, evolutionary & environmental sciences

For a reference copy of the document with all sections, see nature.com/documents/nr-reporting-summary-flat.pdf

Life sciences study design

All studies must disclose on these points even when the disclosure is negative.

Sample size	Cultivation and sequencing experiments were performed in biological triplicates. The consistently high correlation between these replicates (i.e., consistent growth kinetics, cell morphology/structure, and gene expression levels) suggests that this is sufficient.
Data exclusions	No data exclusion was implemented.
Replication	Culture and RNA experiments were performed in triplicate and duplicate, respectively.
Randomization	Randomization not relevant to data collection/analyses in this study
Blinding	The investigators were not blinded to allocation during cultivation-based experiments. No allocation of groups took place in this study (i.e., there was no blinding). Blinding during sample collection and library preparation is not necessary as the output from the sequencing runs is analyzed with an automated pipeline and further displaying and additional analyses were performed in an unbiased manner, with pre-established criteria, and by different persons than those who collected and prepared the samples.

Reporting for specific materials, systems and methods

We require information from authors about some types of materials, experimental systems and methods used in many studies. Here, indicate whether each material, system or method listed is relevant to your study. If you are not sure if a list item applies to your research, read the appropriate section before selecting a response.

Materials & experimental systems

n/a	Involved in the study	
☒	☐	Antibodies
☒	☐	Eukaryotic cell lines
☒	☐	Palaeontology and archaeology
☒	☐	Animals and other organisms
☒	☐	Human research participants
☒	☐	Clinical data
☒	☐	Dual use research of concern

Methods

n/a	Involved in the study	
☒	☐	ChIP-seq
☒	☐	Flow cytometry
☒	☐	MRI-based neuroimaging
