# Supplementary material for: Isolation of a member of the candidate phylum ‘Atribacteria’ reveals a unique cell membrane structure
Source: Nat Commun. 2020 Dec 14;11:6381. doi: 10.1038/s41467-020-20149-5 (PMC7736352; doi:10.1038/s41467-020-20149-5)
Supplement: Supplementary file 2 — Peer Review File [file 41467_2020_20149_MOESM2_ESM.pdf]

**Editorial Note:** This manuscript has been previously reviewed at another journal that is not operating a transparent peer review scheme. The manuscript was considered suitable for publication without further review at *Nature Communications*.
